# Correlation analysis of metabolic characteristics and the risk of metabolic-associated fatty liver disease - related hepatocellular carcinoma

**DOI:** 10.1038/s41598-022-18197-6

**Published:** 2022-08-17

**Authors:** Xuancheng Xie, Mengyao Zheng, Weibo Guo, Ying Zhou, Zhao Xiang, Yuting Li, Jinhui Yang

**Affiliations:** Department of Gastroenterology, Second Affiliated Hospital of Kunming Medical University, Kunming, 650000 China

**Keywords:** Hepatocellular carcinoma, Non-alcoholic fatty liver disease, Risk factors, Metabolic disorders

## Abstract

Metabolic-associated fatty liver disease (MAFLD) is currently the most common chronic liver disease worldwide and the main cause of hepatocellular carcinoma (HCC). To explore the risk factors of MAFLD-HCC, we evaluated the independent and combined effects of metabolic characteristics on the risk of MAFLD-HCC. We retrospectively analyzed 135 MAFLD-HCC patients who were treated at the Second Affiliated Hospital of Kunming Medical University from January 2015 to December 2020 and 135 MAFLD patients as the control group. Independent and joint effects of metabolic traits on the risk of HCC were evaluated. Each metabolic feature was significantly correlated with the increased risk of MAFLD-HCC (*p* < 0.05); obesity had the strongest correlation (adjusted odds ratio [OR] 3.63, 95% confidence interval [CI] 1.99–6.62). In patients with superimposed features, HCC risk was higher with more metabolic features (*p* < 0.05). The correlation between metabolic characteristics and risk of MAFLD-HCC in patients without cirrhosis or advanced fibrosis was basically consistent with the overall analysis. Metabolic characteristics increase the risk of MAFLD-HCC, and the risk is positively correlated with the number of metabolic characteristics. Obesity has the strongest correlation with HCC.

## Introduction

Metabolic-associated fatty liver disease (MAFLD), once known as non-alcoholic fatty liver disease, is a chronic fatty liver disease related to metabolic syndrome, and its incidence has rapidly increased in the past two decades. MAFLD, which now affects 25% of the world’s population and has become the most common chronic liver disease, is considered to be the leading cause of hepatocellular carcinoma (HCC) in developed countries^1,2^. Currently, regional differences in the prevalence of MAFLD are no longer obvious. The prevalence of MAFLD in Asian countries is even higher than in Western Europe and North America, and the onset is gradually occurring at younger ages^3^. However, there is still a lack of specific drugs for the treatment of MAFLD here in China and abroad. Although lifestyle interventions can prevent and treat MAFLD and metabolic cardiovascular risk factors, they are difficult to implement.

HCC is the fifth most common tumor in the world and the third leading cause of cancer-related deaths. Unlike HCC resulting from other causes, the etiology of MAFLD-HCC is unclear, and HCC may also occur in MAFLD patients without cirrhosis^4^. The incidence of MAFLD-HCC is increasing continually, and treatment methods are scarce. Studying the etiology and risk factors of this disease is therefore helpful in the screening, monitoring, and prevention of early HCC. Due to the close relationship between MAFLD and metabolic syndrome, several recent studies have pointed out that in the Western populations, metabolic characteristics, especially risk factors for diseases such as obesity, hypertension, type 2 diabetes mellitus (T2 DM), and dyslipidemia, are closely related to MAFLD-HCC^5,6^. However, the strength and extent of this association have not been clarified. Due to the superposition and interaction of metabolic characteristics and the differences in metabolic characteristics and HCC risk factors among people of different races and geographic locations, the etiology and pathogenesis of MAFLD-HCC are complex^7^. To study the etiology of MAFLD- HCC in Asians and its association with metabolic syndrome, we conducted a retrospective cohort study to evaluate the independent and combined effects of metabolic characteristics on the disease.

## Materials and methods

### Study design and patient population

We retrospectively analyzed 18 to 80-year-old MAFLD-HCC patients who attended the Second Affiliated Hospital of Kunming Medical University from January 2015 to December 2021. The inclusion criteria were patients with MAFLD who were diagnosed with HCC for the first time, and HCC was diagnosed according to the 2010 American Association for the Study of Liver Diseases criteria^8^. The diagnosis of MAFLD was defined by international expert consensus as evidence of liver steatosis with histological biopsy, imaging or blood biomarkers, and included any one of the following three criteria: overweight/obesity, T2 DM, or metabolic syndrome^9^. The exclusion criteria were past history of HCC or other liver diseases that can lead to liver cancer, including viral hepatitis, alcoholic liver disease(alcohol-use disorder defined as consumption of > 3 drinks per day in men and > 2 drinks per day in women, or binge drinking [defined as > 5 drinks in males and > 4 drinks in females, consumed over a 2 h period]^9^), and other rare causes (autoimmune liver disease, primary biliary cirrhosis, hemochromatosis, alpha-1 antitrypsin disease, etc.).

We selected patients who were diagnosed with simple MAFLD (MAFLD without HCC) in 2015 and followed up until December 2021 as the control group. The inclusion criteria were the patient’s visit records for at least 5 years after the diagnosis of MAFLD showed that the patient did not have any other liver diseases. The exclusion criteria were the patient had any other liver diseases during follow-up (including viral hepatitis, alcohol-associated fatty liver disease, drug-induced liver injury and autoimmune hepatitis) or lost to visit. We used random sampling without replacement to select a control cohort that matched the baseline data of the study cohort in terms of gender, age and the presence of fibrosis. Demographic data (age, sex) and biochemical indicators of all patients were recorded at the time of first diagnosis of MAFLD/MAFLD-HCC (surgical admissions). Biochemical indicators included bilirubin, alanine aminotransferase (ALT), aspartate aminotransferase (AST), platelet (PLT), albumin, plasma high-density lipoprotein (HDL), plasma triglycerides, hypersensitive C-reactive protein (CRP) and fasting blood glucose. The body mass index (BMI) was calculated by the formula: weight/height in meters squared (kg/m^2^).

This study was approved by the Ethical Committee of Second Affiliated Hospital of Kunming Medical University (Approval No. Shen-PJ-2020-26). Subjects were entirely informed about the purpose and constraints of this study prior to data collection, and all participants provided their written informed consent. All methods were carried out in accordance with the ethical standards of the responsible committee on human experimentation and with the Declaration of Helsinki.

### Variable specification

Liver cirrhosis was defined as histological or non-invasive elastography showing fibrosis stage 4, portal hypertension syndrome (unexplained splenomegaly or thrombocytopenia, ascites, hepatic encephalopathy, or imaging/endoscopic varicose veins) or radiology showing morphologic features or changes consistent with cirrhosis or portal hypertension. We calculated the Fibrosis-4 (FIB-4) score to define liver fibrosis severity in our study, which was calculated by the formula: age (years) × AST (U/L)/ [PLT (10^9^/L) × ALT^1/2^ (U/L)], We used cut-off > 1.3 to define fibrosis and cut-off ≥ 2.67 to define high FIB-4 because it was shown to be highly predictive of the presence of advanced fibrosis or cirrhosis in patients with MAFLD^10^. The main exposure metabolic disorders observed included obesity/overweight (BMI ≥ 23 kg/m^2^ in Asians), type 2 diabetes, prediabetes, hypertension, and dyslipidemia. Dyslipidemia included hypertriglyceridemia (plasma triglycerides ≥ 1.70 mmol/L) and low HDL (plasma HDL < 1.0 mmol/L). Prediabetes was defined as fasting blood glucose levels of 5.6–6.9 mmol/L or 2-h post-load blood glucose levels of 7.8–11.0 mmol/L. T2 DM and hypertension were defined as fasting blood glucose ≥ 7.0 mmol/L or 2-h postprandial blood glucose ≥ 11.1 mmol/L or oral hypoglycemic drugs, insulin, and blood pressure ≥ 130/85 mmHg or specific drug treatment, respectively^9^. Metabolic characteristics of all patients were assessed using data at the time of first diagnosis of MAFLD-HCC/MAFLD.

### Statistical analysis

Descriptive statistics are reported as percentage for categorical variables and mean ± SD or median (interquartile range) for continuous variables. Continuous variables with a normal distribution were compared by *t* test, and continuous variables with a non-normal distribution were compared by the Wilcoxon rank sum statistic. Categorical variables were compared using the chi-square test. The chi-square test was also used to analyze the differences in different factors between the two groups of patients, and then single-factor and multivariate logistic regression analyses were used to determine whether the independent and combined effects of these factors were significantly different.

It is common to have multiple metabolic characteristics superimposed in one patient. To analyze the combined effects of different metabolic variables, in the subgroup analysis, we modeled the metabolic characteristic variables as additive indicators (as the number of traits) and analyzed the number of traits on the risk of MAFLD-HCC. At last, we conducted multivariable models adjusting for waist circumference and CRP > 2 mg/L to determine the independent effect of metabolic characteristic on the risk of MAFLD-HCC.

### Sensitivity analyses

A substantial proportion of MAFLD-HCC occurs in patients without cirrhosis, and it is unclear whether metabolic characteristics have the same effect in this subset of patients. We screened all MAFLD-HCC patients without cirrhosis in this study and used the same model as the overall analysis to analyze the independent and combined effects of metabolic characteristics and HCC risk. In addition, metabolic characteristics are also associated with higher risk of advanced fibrosis (even if not cirrhosis) and this could be a major mediator, so we also screened all MAFLD-HCC patients without advanced liver fibrosis (FIB-4 ≥ 2.67) for analysis. We investigated whether the influence of metabolic characteristics among the two portions of the patients and the overall cohort was different.

All statistical analyses were performed using SPSS, version 19.0.0.1 (IBM SPSS, 2010, Chicago, IL, USA). All *p* values were two-tailed, and results were considered statistically significant at *p* < 0.05.

## Results

### Patient characteristics

**Figure 1:**
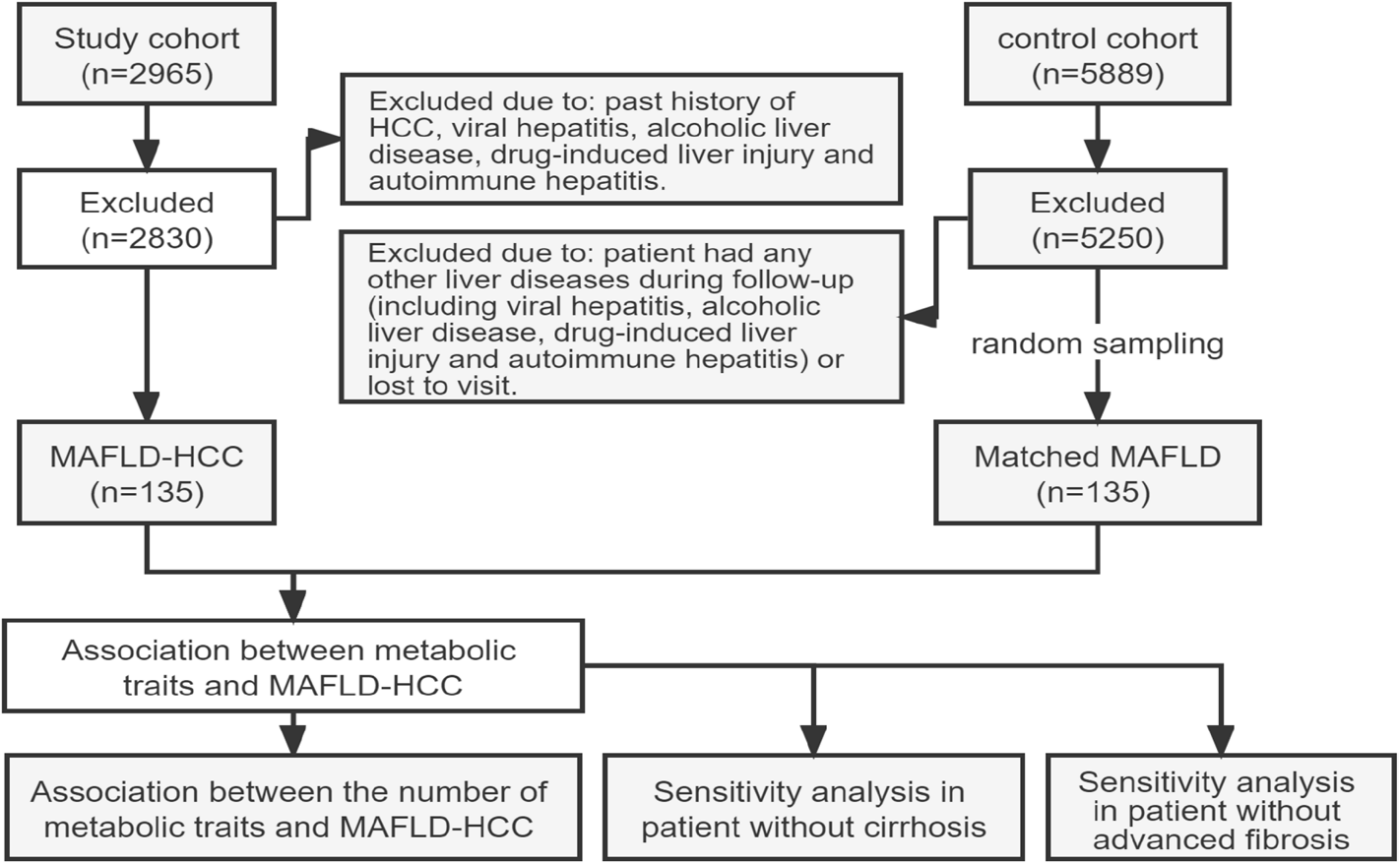
Flowchart of the study. *MAFLD* metabolic-associated fatty liver disease, *MAFLD-HCC* metabolic-associated fatty liver disease related hepatocellular carcinoma.

A total of 2965 patients with HCC were included in this study, and 135 MAFLD-HCC patients fulfilled study criteria. A total of 5889 patients with MAFLD were included in the control cohort and 5250 patients were excluded due to the exclusion criteria. Then, 135 matched patients with only MAFLD were randomly selected as a control cohort (Fig. 1). There was no significant difference in the average age or sex/fibrosis ratio between the two groups of patients (*p* > 0.05). Among MAFLD-HCC patients, 26 had liver cirrhosis (19.3%), 31 had T2 DM (23%), 46 had prediabetes (34.1%), 110 had obesity (81.5%), 74 had hypertension (54.8%), and 38 (28.1%) had advanced fibrosis. There were 91 patients (61.4%) with dyslipidemia, of which 62 (45.9%) had hypertriglyceridemia and 64 (47.4%) had low HDL. Compared with the control cohort, patients with MAFLD-HCC had a higher prevalence of prediabetes, obesity, hypertension, dyslipidemia and advanced fibrosis, and the difference was statistically significant (*p* < 0.05), However, there was no statistically significant difference in the prevalence of T2 DM (*p* = 0.224). The biochemical indicators of the two groups of patients revealed worse liver function in patients with MAFLD- HCC (*p* < 0.05) (Table 1).

**Table 1:** Baseline characteristics of patients with MAFLD and MAFLD-HCC.

Characteristic	HCC (n = 135)	Non-HCC (n = 135)	*p*
Age (years)	57.26 ± 12.59	59.52 ± 13.69	0.159
**Sex**			
Male	82 (60.7%)	74 (54.8%)	0.324
Female	53 (39.3%)	61 (45.2%)	
Cirrhosis	26 (19.3%)	0 (0%)	-
Prediabetes	46 (34.1%)	30 (22.2%)	0.030
Type II diabetes	31 (23.0%)	23 (17.0%)	0.224
Prediabetes or Type II diabetes	77 (57.0%)	53 (39.3%)	0.003
**waist circumference(cm)**			
Male	93.16 ± 14.56	97.58 ± 15.91	0.072
Female	85.11 ± 16.11	81.82 ± 16.66	0.287
BMI	24.50 ± 2.96	24.96 ± 3.31	0.229
BMI ≥ 23 kg/m^2^	110 (81.5%)	75 (55.6%)	< 0.001
Hypertension	74 (54.8%)	48 (35.6%)	0.001
**Dyslipidemia**			
Dyslipidemia	91 (67.4%)	61 (45.2%)	< 0.001
HDL < 1.0 mmol/L	64 (47.4%)	45 (33.3%)	0.018
Triglycerides ≥ 1.7 mmol/L	62 (45.9%)	48 (35.6%)	0.083
Albumin (g/L)	39.4 ± 6.94	41.36 ± 3.78	0.002
Bilirubin (μmol/L)	17.1[10.7, 39.9]	13.2 [10.2, 16.9]	< 0.001
ALT (U/L)	39.0 [25.0, 63.0]	24.0 [16.0, 40.0]	< 0.001
AST (U/L)	35.0 [26.0, 59.0]	23.0 [19.0, 29.0]	< 0.001
PLT (10^9^/L)	214.33 ± 92.03	218.46 ± 60.18	0.663
CRP (mg/L)	16.03 ± 12.89	4.93 ± 4.86	< 0.001
CRP > 2 mg/L	125 (92.6%)	102 (75.6%)	< 0.001
FIB-4	2.49 ± 2.37	1.50 ± 0.92	< 0.001
FIB-4 > 1.3	91 (67.4%)	82 (60.7%)	0.254
FIB-4 ≥ 2.67	38 (28.1%)	8 (5.9%)	< 0.001

*BMI* body mass index, *HDL* high-density lipoprotein, *ALT* alanine aminotransferase, *AST* aspartate aminotransferase, *PLT* platelet, *CRP* hypersensitive C-reactive protein, *FIB-4* fibrosis-4.

### Independent correlation of metabolic characteristics

We conducted an independent correlation analysis for each significantly different metabolic characteristic, and the results showed that each characteristic was significantly related to the increased risk of MAFLD-HCC (*p* < 0.05). The risk of MAFLD-HCC in patients with obesity, prediabetes or T2 DM, hypertension, and dyslipidemia was 3.5 times (95% confidence interval [CI] 2.03–6.11), 2.1 times (95% CI 1.26–3.34), 2.2 times (95% CI 1.35–3.59), and 2.5 times (95% CI 1.53–4.11) greater than that of patients without the disease (Table 2).

**Table 2:** Association between metabolic traits and MAFLD-HCC on univariate and multivariate logistic regression analysis.

Characteristic	Crude OR (95% CI)	*p*	Adjusted OR (95% CI)	*P*
Waist circumference	1.00 (0.99–1.02)	0.822	–	–
CRP > 2 mg/L	4.04 (1.90–8.60)	< 0.001	3.69 (1.62–8.43)	0.002
Prediabetes or Type II diabetes	2.05 (1.26–3.34)	0.004	2.01 (1.17–3.44)	0.012
BMI ≥ 23 kg/m^2^	3.52 (2.03–6.11)	< 0.001	3.63 (1.99–6.62)	< 0.001
Hypertension	2.20 (1.35–3.59)	0.002	2.25 (1.30–3.88)	0.004
Dyslipidemia	2.51 (1.53–4.11)	< 0.001	2.58 (1.49–4.46)	0.001

*FIB-4* fibrosis-4, *BMI* body mass index.

**Figure 2:**
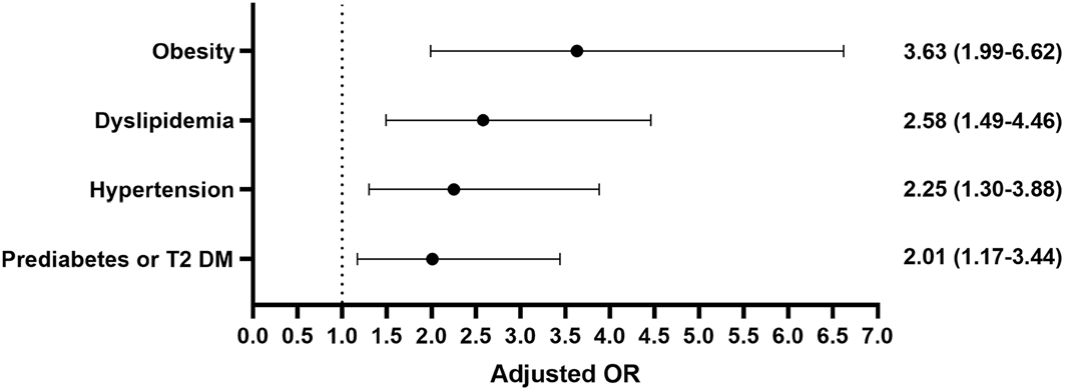
Adjusted associations between metabolic traits and MAFLD-HCC on multivariate logistic regression analysis. *T2 DM* type II diabetes.

In the multivariate model, the obesity, dyslipidemia, and hypertension patients had an adjusted increased risk of disease compared with those without disease: 3.6 times (95% CI 1.99–6.62), 2.6 times (95% CI 1.49–4.46), and 2.3 times (95% CI 1.30–3.88) increased risk compared with that of patients without disease, respectively. The risk of patients with prediabetes or T2 DM after adjustment was greater than that of patients without disease by 2 times (95% CI 1.17–3.44), which was decreased (Fig. 2).

### Joint correlation of metabolic characteristics

A ubiquitous superposition of metabolic characteristics was observed in patients with MAFLD-related HCC. The proportions of patients with two metabolic characteristics were 56.3% (obesity and dyslipidemia), 41.5% (obesity and hypertension), 34.8% (prediabetes or T2 DM and hypertension), 47.4% (prediabetes or T2 DM and obesity), 37.8% (hypertension and dyslipidemia), and 42.2% (prediabetes or T2 DM and dyslipidemia). The proportions of patients with three metabolic characteristics at the same time were 29.6% (obesity, hypertension, and dyslipidemia), 15.6% (prediabetes or T2 DM, hypertension, and dyslipidemia), 11.9% (obesity, prediabetes or T2 DM, and hypertension), and 14.1% (obesity, prediabetes or T2 DM, and dyslipidemia). Patients with four characteristics at the same time accounted for 22.2% of the study group. Compared with the control cohort, all data differences were statistically significant (*p* < 0.05) (Table 3).

**Table 3:** Types of metabolic traits of patients with MAFLD and MAFLD-HCC. T2 DM, Type II diabetes.

Characteristics	HCC (n = 135)	Non-HCC (n = 135)	χ^2^	*p*
Obesity and dyslipidemia	76 (56.3%)	29 (21.5%)	34.426	< 0.001
Obesity and hypertension	56 (41.5%)	29 (21.5%)	12.517	< 0.001
Prediabetes/T2 DM and hypertension	47 (34.8%)	14 (10.4%)	23.063	< 0.001
Prediabetes/T2 DM and obesity	64 (47.4%)	27 (20.0%)	22.692	< 0.001
Hypertension and dyslipidemia	51 (37.8%)	24 (17.8%)	13.456	< 0.001
Prediabetes/T2 DM and dyslipidemia	57 (42.2%)	21 (15.6%)	23.365	< 0.001
Obesity, hypertension, and dyslipidemia	40 (29.6%)	10 (7.4%)	23.388	< 0.001
Prediabetes/T2 DM, hypertension, dyslipidemia	21(15.6%)	5 (3.7%)	10.895	0.001
Obesity, prediabetes/T2 DM, hypertension	16 (11.9%)	4 (3.0%)	7.776	0.005
Obesity, prediabetes/T2 DM, dyslipidemia	19 (14.1%)	9 (6.7%)	3.985	0.046
Prediabetes/T2 DM, obesity, hypertension, and dyslipidemia	30 (22.2%)	2 (1.5%)	27.794	< 0.001

**Figure 3:**
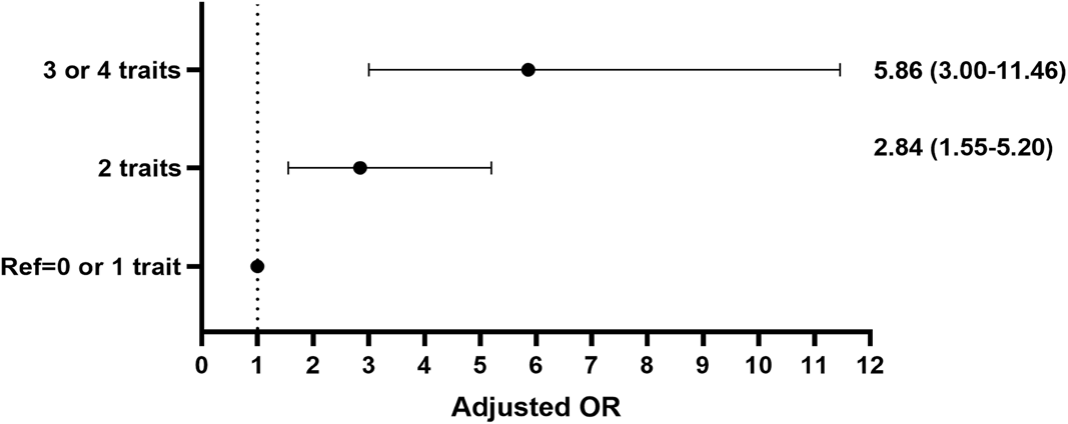
Adjusted associations between the number of metabolic traits and MAFLD-HCC on multivariate logistic regression analysis.

In the univariate model, the correlation analysis between the superimposition of each metabolic feature and the risk of MAFLD-HCC showed that all patients with superimposed characteristics had higher risk than those without superimposition (*p* < 0.05). In the multivariate model, we analyzed the association between the number of metabolic traits and the risk of MAFLD-HCC. Due to the limited number of samples, we divided patients as with 0 or 1 trait group, 2 traits group and 3 or 4 traits group. The proportion of each group was 15.6% (0 or 1 trait), 29.6% (2 traits) and 54.8% (3 or 4 traits) in the study cohort and 39.3% (0 or 1 trait), 37.8% (2 traits) and 23.0% (3 or 4 traits) in the control cohort. Compared with patients with one or no traits, the risk of MAFLD-HCC increased to 2.8 (95% CI 1.55–5.20) and 5.9 times (95% CI 3.00–11.46) for having 2 and 3/4 traits, respectively (Table 4, Fig. 3).

**Table 4:** Association between the number of metabolic traits and MAFLD-HCC on multivariate logistic regression analysis. HCC, hepatocellular carcinoma.

	0 or 1 trait	2 traits	3 or 4 traits
HCC (n = 135)	21 (15.6%)	40 (29.6%)	74 (54.8%)
Non-HCC (n = 137)	53 (39.3%)	51 (37.8%)	31 (23.0%)
Adjusted OR (95% CI)	1 [Reference]	2.84 (1.55–5.20)	5.86 (3.00–11.46)
*P*	–	< 0.001	< 0.001

*Adjusted for CRP > 2 mg/L and Waist circumference.

### Sensitivity analyses

In this study, a total of 109 patients were diagnosed with HCC without cirrhosis, and only 19.3% of patients were diagnosed with liver cirrhosis and HCC. The analysis of the relationship between metabolic characteristics and risk of disease in patients with MAFLD-HCC without liver cirrhosis showed that patients with prediabetes or T2 DM, obesity, hypertension, and dyslipidemia had 2.4 times (95% CI 1.35–4.23), 3.9 times (95% CI 2.02–7.40), 1.8 times (95% CI 1.02–3.24) and 2.6 times (95% CI 1.46–4.66) higher risk than those without the disease; after adjustment, the differences were statistically significant (*p* < 0.05) (Table 5).

**Table 5:** Associations between metabolic traits and MAFLD-HCC in patients without cirrhosis on univariate and multivariate logistic regression analysis.

Characteristic	Crude OR (95% CI)	*p*	Adjusted OR (95% CI)	*P*
Waist circumference	1.00 (0.99–1.02)	0.687	–	–
CRP > 2 mg/L	4.09 (1.80–9.27)	0.001	3.56 (1.46–8.67)	0.005
Prediabetes or Type II diabetes	2.38 (1.42–3.98)	0.001	2.39 (1.35–4.23)	0.003
BMI ≥ 23 kg/m^2^	3.79 (2.08–6.91)	< 0.001	3.86 (2.02–7.40)	< 0.001
Hypertension	1.85 (1.10–3.09)	0.020	1.82 (1.02–3.24)	0.042
Dyslipidemia	2.46 (1.46–4.15)	0.001	2.61 (1.46–4.66)	0.001

*FIB-4* fibrosis-4, *BMI* body mass index.

A total of 97 patients in the study cohort and 127 in the control cohort were diagnosed without advanced fibrosis. The analysis of the relationship between metabolic characteristics and risk of disease in patients with MAFLD-HCC without advanced fibrosis showed that patients with prediabetes or T2 DM, obesity, hypertension, and dyslipidemia had 3.1 times (95% CI 1.66–5.85), 4.2 times (95% CI 2.08–8.63), 3 times (95% CI 1.58–5.65) and 3 times (95% CI 1.59–5.69) higher risk than those without the disease; after adjustment, the differences were statistically significant. (*p* < 0.05) (Table 6).

**Table 6:** Associations between metabolic traits and MAFLD-HCC in patients without high FIB-4 on univariate and multivariate logistic regression analysis.

Characteristic	Crude OR (95% CI)	*p*	Adjusted OR (95% CI)	*P*
Waist circumference	1.00 (0.98–1.01)	0.593	–	–
CRP > 2 mg/L	3.75 (1.64–8.57)	0.002	3.36 (1.32–8.57)	0.011
Prediabetes or Type II diabetes	2.73 (1.59–4.72)	< 0.001	3.11 (1.66–5.85)	< 0.001
BMI ≥ 23 kg/m^2^	3.96 (2.11–7.42)	< 0.001	4.24 (2.08–8.63)	< 0.001
Hypertension	2.65 (1.54–4.58)	< 0.001	2.99 (1.58–5.65)	0.001
Dyslipidemia	2.93 (1.67–5.14)	< 0.001	3.01 (1.59–5.69)	0.001

*FIB-4* fibrosis-4, *BMI* body mass index.

## Discussion

The global prevalence of MAFLD has risen from 15 to 25% over the past 10 years, and this trend is expected to continue^11^. The risk of HCC, the most serious complication of MAFLD, is also increasing. At present, MAFLD is considered as the most common risk factor for liver cancer in the United States and Japan, and MAFLD-HCC is considered an emerging indication for liver transplantation^12,13^. However, data from large-scale studies of the incidence and risk of MAFLD-HCC in China are lacking. MAFLD is the manifestation of metabolic syndrome in the liver. Numerous studies have confirmed that metabolic characteristics are closely related to the development of HCC. Particularly patients without cirrhosis, obesity and T2 DM are considered independent risk factors for the development of HCC^14,15^. In this study, we found that prediabetes or T2 DM, obesity, hypertension, and hyperlipidemia are all individually or in combination associated with an increased risk of HCC, and this risk is positively correlated with the number of metabolic characteristics.

As there was no statistically significant difference in the prevalence of T2 DM in the two study cohorts in the independent and joint correlation analyses of metabolic characteristics, we selected prediabetes as an alternative metabolic characteristic based on the diagnostic criteria of MAFLD and confirmed that it has a strong correlation with HCC risk. In the multivariate model, we entered prediabetes and T2 DM as one factor for analysis and found that obesity was the factor most strongly associated with risk of HCC progression; this risk was 3.6 times that of non-obese individuals. Several studies have noted that although obesity is associated with increased risk of many cancers, it has the strongest correlation with increased risk of HCC, which is consistent with our results^16,17^.

In the joint correlation analysis of metabolic characteristics, the more metabolic characteristics the patient have, the higher risk of MAFLD-HCC. The risk of MAFLD-HCC in patients with 3 or 4 traits was 5.9 times (adjusted OR = 7.49, 95% CI 3.00–11.46) greater than that of patients with 0 or 1 trait, demonstrating that the number of metabolic characteristics is positively correlated with disease risk. Our results are basically consistent with those of previous studies in which higher burden of coexisting metabolic traits was linked with higher risk of HCC, and each additional metabolic trait increased the risk of HCC in patients with MAFLD^18^.

The results of the current study indicate that a considerable proportion of MAFLD-HCC appears in patients without cirrhosis^19,20^. In our study, this proportion was 80%, slightly higher than that reported from studies in other parts of the world. In the correlation analysis of metabolic characteristics in this group of patients, the correlation between obesity, prediabetes or T2 DM, and dyslipidemia and HCC risk was slightly higher than that in the overall analysis, whereas the correlation between hypertension and HCC risk was slightly lower, with obesity remaining the most strongly correlated risk factor (adjusted OR = 3.86, 95% CI 2.02–7.40). In addition, the proportion of MAFLD-HCC patients without advanced fibrosis was 72%. In the correlation analysis of metabolic characteristics in this group of patients, the correlation between all the traits and HCC risk was slightly higher than that in the overall analysis, and obesity conferred the highest risk of MAFLD-HCC as well (adjusted OR = 4.24, 95% CI 2.08–8.63). Overall, our results in both analyses of this part were consistent with the overall analysis.

Surveillance of MAFLD-HCC is very challenging. The current guidelines of European Association for the Study of the Liver only recommend monitoring patients with MAFLD-cirrhosis. It is recommended that abdominal ultrasound and serum alpha-fetoprotein examinations be performed every 6 months^21^. However, the guidelines ignore the occurrence of HCC in patients without cirrhosis, and there is no precise screening recommendation. Our research provides a basis for accurate screening and risk stratification of MAFLD-HCC. According to our research results, HCC surveillance is critical for patients with multiple metabolic characteristics. In addition, data regarding related metabolic factors such as obesity, prediabetes or T2 DM, dyslipidemia, and hypertension, as confirmed by our research, are objective and easy to obtain, which can provide a scientific basis for the establishment of a cost-effective accurate surveillance tool for HCC. Simultaneously, these metabolic factors could also be used as an important target of secondary prevention to delay the progress of MAFLD-HCC.

This study has several limitations. First, this was a retrospective analysis, and the retrospective, non-randomized design could introduce selection bias. Second, there may be problems with the selection of the patient metabolic factors. For example, we only selected biochemical indicators when a patient was first diagnosed with HCC, which could have introduced errors, and there are obesity markers that are more sensitive than BMI. Third, considering that patients in the control group also have risk factors of HCC, a long-term follow-up is needed to observe the prognosis and outcome of these patients. Fourth, limited by retrospective analysis, we missed homeostasis model assessment of insulin resistance score of patients in the two cohorts, which is one of the seven diagnostic criteria for metabolic dysregulation. Finally, our research did not involve treatment methods or patient prognosis. Our proposed strategy for secondary prevention goals thus requires prospective risk reduction trials to demonstrate its effectiveness.

In summary, our study found that metabolic characteristics increase the risk of MAFLD-HCC, and this risk is positively correlated with the number of metabolic characteristics. Regardless of the presence or absence of cirrhosis or advanced fibrosis, obesity has the strongest correlation with the risk of MAFLD-HCC. Our results indicate that monitoring of prediabetes or T2 DM, hypertension, dyslipidemia, and obesity can be used to comprehensively assess the risk of HCC, which can in turn facilitate the establishment of reasonable, cost-effective risk stratification and precise screening strategies.

## Data Availability

The datasets used and analysed during the current study available from the corresponding author (Jinhui Yang, email: yangjinhuikmmc@163.com) on reasonable request.
